# Common neural correlates of disgust processing in childhood maltreatment and peer victimisation

**DOI:** 10.1192/bjo.2024.767

**Published:** 2024-10-28

**Authors:** Lena Lim, Katya Rubia, Steve Lukito

**Affiliations:** Singapore Institute for Clinical Sciences, Agency for Science, Technology and Research (A*STAR), Singapore; and Department of Child & Adolescent Psychiatry, Institute of Psychiatry, Psychology & Neuroscience, King's College London, UK; Department of Child & Adolescent Psychiatry, Institute of Psychiatry, Psychology & Neuroscience, King's College London, UK

**Keywords:** Childhood trauma, early-life stress, child abuse, emotion processing, peer bullying

## Abstract

**Background:**

Childhood maltreatment and peer victimisation are common sources of early-life interpersonal stress. Childhood maltreatment is associated with atypical frontolimbic emotion processing and regulation, and increased vulnerability for self-harm/suicide. However, few studies have compared the neurofunctional correlates between caregiver- versus peer-inflicted mistreatment.

**Aims:**

We compared the alterations of neurofunctional correlates of facial emotion processing in youths exposed to childhood maltreatment or peer victimisation, and explored their associations with self-harm.

**Method:**

Functional magnetic resonance imaging data were collected from 114 age- and gender-matched youths (39 childhood maltreatment, 37 peer victimisation and 38 controls) during an emotion discrimination task. Region-of-interest (amygdala, insula) and whole-brain analyses were conducted.

**Results:**

Groups differed significantly during disgust processing only. Both groups had lower activation in the right amygdala and bilateral posterior insula than controls; left insular underactivation was furthermore related to increased self-harm in maltreated youths. Compared with controls, at the whole-brain level, both groups also had underactivation in a cluster of bilateral limbic-thalamic-striatal, precuneus/posterior cingulate, temporal, fusiform/lingual and cerebellar regions, which was negatively associated with emotional problems in controls, as well as a cluster of somatosensory regions associated with increased self-harm in maltreated youths.

**Conclusions:**

Early-life interpersonal stress from caregivers or peers is associated with common underactivation of limbic-thalamic-striatal, precuneus/posterior cingulate and somatosensory regions during disgust processing. The hypoactivation of key emotion and sensory processing and self-referential brain regions could be a potential suppressive mechanism to cope with the aversive emotion; however, it may also entail increased risk of affective psychopathology in seemingly healthy youths.

Emerging research underscores the influence of childhood trauma and early-life stress on the brain development of young people. Childhood maltreatment, which includes physical, sexual and emotional abuse and neglect, is a global issue with prevalence of 13–36%.^1^ Childhood maltreatment is associated with a wide range of psychosocial and developmental difficulties, including atypical emotion processing.^2^ Within the familial setting, repeated exposure to harsh caregiving during childhood heightens the child's sensitivity to negative socioemotional signals and adversely influences the development of neural pathways underlying emotion processing.^3^ Converging evidence indicates that childhood maltreatment is one of the strongest risk factors for psychiatric disorders, including depression, anxiety, post-traumatic stress disorder and self-harm/suicide,^4^ where the ability to process aversive emotions seems particularly affected across disorders.^5^

## Childhood maltreatment

The psychopathological outcomes associated with childhood maltreatment may be mediated by alterations in neural mechanisms underlying emotion processing. Structural magnetic resonance imaging (MRI) and review studies show that childhood maltreatment is associated with grey matter volume (GMV) abnormalities in several stress-susceptible and emotion processing brain regions, including the orbitofrontal cortex, limbic, insula and cerebellum.^2,6^ Our meta-analysis of structural MRI studies found that childhood maltreatment is associated with GMV reduction in orbitofrontal cortex-limbic-temporal regions that mediate top-down affect control, and with GMV reduction in pre-/postcentral gyri that mediate sensory functions.^7^ Our recent meta-analysis of structural connectivity in childhood maltreatment underscores the involvement of corticolimbic, frontostriatal and occipital visual pathways in the biopsychological consequences of childhood maltreatment, where diminished structural integrity of these circuitries may hinder normal emotional and sensory functioning and increase vulnerability to psychopathology.^8^ At the neurofunctional level, a meta-analysis of functional MRI (fMRI) studies of emotional face processing reported increased cortical and limbic activations, including the amygdala/parahippocampal, insula and superior temporal gyri, among individuals exposed to childhood maltreatment relative to controls.^9^ Atypical limbic reactivity to negative emotional faces has been consistently reported across studies in childhood maltreatment. For instance, viewing of threat-related (angry and/or fearful) emotional faces has been associated with amygdala over-reactivity^3,10^ or under-reactivity^11^ in maltreated individuals, which has been interpreted as hypervigilance or avoidance to threats, respectively. The childhood maltreatment-related atypical amygdala reactivity to negative emotions has been further proposed to mediate the development of anxiety and depression.^12^

## Peer victimisation

Another source of early-life stress is navigating through peer relationships. Although peers may constitute a vital source of social support outside the familial settings, they can also be a significant cause of interpersonal stress. Peer victimisation, which has a prevalence rate of 30% worldwide,^13^ is characterised by repetitive aggressive behaviour engaged to cause harm to the victim.^14^ It includes overt confrontation, ostracism, relational and reputational aggression. Peer victimisation is associated with poor school performance and the development of psychiatric problems, including anxiety, depression and self-harm/suicide.^15^

Most fMRI studies in peer victimisation have focused on social exclusion and reported enhanced activation of limbic and cortical regions, including the amygdala, insula, anterior cingulate and medial prefrontal cortex among bullied adolescents and young adults compared with controls.^16^ Notably, fMRI studies involving emotional face processing in this population is relatively limited. Recent studies found that greater amygdala reactivity to angry and fearful faces predicted higher levels of relational victimisation in healthy adolescents,^17^ peer victimisation during mid-adolescence was associated with augmented amygdala response toward fear and angry faces in young adulthood,^11^ and history of victimisation among female adolescents with high relative to low rejection sensitivity is associated with higher amygdala-ventrolateral prefrontal cortex connectivity when viewing emotional faces indicating lower effectiveness of emotional regulation.^18^ Therefore, like childhood maltreatment, peer victimisation may also be associated with atypical limbic reactivity toward negative emotional faces.

Research has shown that atypical emotion processing increases self-harm/suicide risk.^19^ Given that self-harm is often executed as an affect-regulation strategy to ease intense negative emotions,^20^ individuals who experienced childhood interpersonal stress may self-harm to cope with the persistent pain of relational rejections. Furthermore, individuals who had attempted suicide demonstrated specific alterations in the recognition of facial disgust compared with patients with depression and healthy controls, which may impair their ability to interact effectively, thereby intensifying the risk of interpersonal conflict and suicide.^19^

The field has made significant progress in documenting the neurobiological correlates of childhood maltreatment, but research investigating neural alterations in peer victimisation has been comparatively limited. Furthermore, childhood maltreatment and peer victimisation may have unique and/or additive effects on the development of maladaptive cognitive structures and psychological maladjustment. For instance, a retrospective study of young adults found that parental emotional abuse (controlling for peer verbal victimisation) predicted dysfunctional attitudes, but not cognitive style; whereas peer victimisation (controlling for parental abuse) predicted cognitive style, but not dysfunctional attitudes.^21^ A longitudinal study of community youths reported that harsh parenting and peer victimisation, taken together or separately, predicted changes in youths’ negative and positive self-cognitions and depressive symptoms, and harsh parenting exhibited incremental importance over and above peer victimisation on youths’ self-cognitions.^22^ Hence, given that childhood maltreatment and peer victimisation may have differential effects on mental health outcomes, and maltreated children are at increased risk of subsequent bullying by peers possibly via altered neurocognitive functioning,^23^ it is imperative that studies examine bullying from peers in the absence of harsh caregiving and *vice versa*, to elucidate the distinctive neural effect of peer victimisation and childhood maltreatment.

Evidence suggests that childhood maltreatment and peer victimisation are associated with atypical limbic reactivity to emotional faces. However, these two distinct experiences have not been investigated within a single study. Therefore, the present study examined the associations between neurofunctional alterations during processing of dynamic facial expressions and early-life interpersonal stress from caregivers (childhood maltreatment) and peers (peer victimisation) in youths by conducting both region-of-interest (ROI) analysis of key emotion processing regions (amygdala and insula) and whole-brain analysis in community youths free from psychopathology, medication and drug misuse. We hypothesised that both the childhood maltreatment and peer victimisation groups would show greater activation than controls, particularly in the limbic regions, during negative emotion processing, and that the atypical reactivity would be associated with greater self-harm. To examine the specificity of the association with the nature of early-life interpersonal stress, we controlled for the timing and duration of exposure to early-life stress, as well as the number of recent stressors experienced.

## Method

### Participants

Right-handed youths aged 17–21 years were recruited from the community via advertisement. Exclusion criteria were childhood sexual abuse, drug misuse, current/past psychiatric disorders, psychotropic medications, neurological abnormalities/brain injuries, intellectual disabilities, bullying perpetration and MRI contraindications. We first conducted a thorough pre-screening interview via phone to assess the study eligibility criteria and early-life stressful experiences before the age of 16 years, where potential participants were first asked if they had any of the exclusion characteristics listed, as well as ‘Have you been bullied by peers?’ and ‘Have you experienced harsh treatment/parenting from your caregiver(s)?’. Severity of the early-life stressful experiences were assessed using the Childhood Trauma Questionnaire (CTQ),^24^ Revised-Peer Experiences Questionnaire (rPEQ)^25^ and European Cyberbullying Intervention Project Questionnaire (ECIPQ).^26^ Information on the age onset and duration of the early-life stressful experiences were collected with the two questions: ‘How old were you when you first experienced the harsh treatment from the caregiver(s) or peer(s)?’ and ‘For how long did you experience the harsh treatment from the caregiver(s) or peer(s)’. Inclusion criteria for the childhood maltreatment group were non-sexual maltreatment from caregivers scoring above the cut-off for moderate severity on at least one of the CTQ subscales, but did not experience bullying from peers (answering ‘No’ to the bully-experience question above and scoring ‘Never’ or ‘Once or twice’ on all of the rPEQ/ECIPQ items). Inclusion criteria for the peer victimisation group were frequently bullied by peers (answering ‘Yes’ to the bully-experience question above and indicating at least ‘A few times’ on at least one rPEQ/ECIPQ item), but without a history of maltreatment from caregivers (scoring below the cut-offs for none/low severity on all of the CTQ subscales). The control group did not experience maltreatment from caregivers or bullying from peers (meeting the same criteria as above). Interested volunteers meeting the study criteria were invited to participate, whereas those unsuitable were notified and their information was deleted immediately. A total of 117 youths (39 childhood maltreatment, 39 peer victimisation and 39 controls) participated in the study.

All participants and their legal guardians provided written informed consent. The authors assert that all procedures contributing to this work comply with the ethical standards of the relevant national and institutional committees on human experimentation and with the Helsinki Declaration of 1975, as revised in 2008. All procedures involving human participants were approved by Nanyang Technological University Singapore Institutional Review Board (approval number IRB-2018-01-025) and all MRI scans were reviewed by a neuroradiologist (Supplementary Material available at https://doi.org/10.1192/bjo.2024.767).

### Study design and procedure

The study consisted of a face-to-face interview and an MRI session that took place within a 1-week period. During the interview session, all participants completed the following: DSM-5 Level-1 Cross-Cutting Symptom Measure and Kiddie Schedule for Affective Disorders and Schizophrenia Present and Lifetime Version (KSADS-PL) interviews for psychopathology, Strengths and Difficulties Questionnaire (SDQ),^27^ Beck Depression Inventory (BDI),^28^ Beck Anxiety Inventory (BAI)^29^ and the Negative and Positive Affect Scale (NAPAS).^30^ The Childhood Experience of Care and Abuse interview^31^ was used to corroborate the CTQ. IQ was assessed using the Wechsler Abbreviated Scale of Intelligence.^32^ Socioeconomic status was measured with six items (on parental educational level, housing size and type) from the Family Affluence Scale.^33^ Self-harming behaviour was assessed with the Self-Harm Inventory (SHI).^34^ Finally, recent stressful life events (RSLE) was assessed using common stressors adapted from the Life Event Questionnaire for Adolescents,^35^ where participants rated the 12-month incidence and distress level of each stressor. A total RSLE score was calculated by summing the number of items that were rated as quite or very stressful. In the present study, the internal consistency of the questionnaires ranged from 0.88 to 0.93.

### fMRI paradigm: emotion discrimination task

The fMRI task was adapted from our previous emotion discrimination task.^3^ In essence, participants were shown 1 s video clips of six actors (three males) displaying disgust, happy, fear, angry or neutral facial expressions. Blocks of stimuli (12 s) of each emotion were interspersed with a fixation baseline condition (6 s). Each emotion was presented in a block of 6 × 1 s stimuli, with each stimulus followed by a 1 s gap. Each emotion block was repeated five times in a pseudo-random order, and neutral was repeated six times. Participants were instructed to identify each clip as positive, negative or neutral.

### fMRI data acquisition and analysis

Data acquisition, preprocessing and first-level analysis are described in the Supplementary Material. Data were processed and analysed with SPM12, version 7771 (Statistical Parametric Mapping for Windows; Wellcome Centre for Human Neuroimaging, London, UK; see https://www.fil.ion.ucl.ac.uk/). Participants were removed from further analysis if movement away from the first collected volume exceeded 3 mm of displacement or 3 degrees of rotation in any direction. For second-level analyses, contrast images from the first level were used to conduct full factorial whole-brain analysis comparing activation across the three groups for each negative emotion (disgust, fear, anger), contrasted with happy. Given that individuals with early trauma tend to perceive neutral faces as negative and since the neutral condition did not contain the same amount of facial movement as the emotion conditions, the happy condition is thus a better-matched contrast as it controlled for motion perception.^3^ The use of happy instead of a neutral condition as a comparative contrast is also a common practice in previous fMRI studies of emotion processing.^3,36^ RSLE, age onset and duration of early-life stress were included as covariates. Blood oxygenation level dependent (BOLD) responses are reported using a family-wise error rate-corrected cluster threshold of *P <* 0.05. Additionally, mask(s) of significant cluster(s) in the whole-brain analysis were created in SPM12 and data were extracted with MarsBaR ROI toolbox for SPM12 (http://marsbar.sourceforge.net/) for subsequent exploratory correlational analyses with psychological measures (SHI, BAI, BDI, SDQ) within each group, using Pearson/point-biserial correlations. For the ROI analysis, the Neuromorphometrics atlas within SPM12 was used to create the ROI masks (amygdala, anterior insula, posterior insula; Supplementary Fig. 1), and data were extracted using MarsBaR for group comparisons with analysis of covariance (ANCOVA), controlling for RSLE, age onset and duration of early-life stress. As the ROIs examined were chosen *a priori* based on the literature, no adjustment for multiple comparisons was made.

### Statistical analyses of demographic and performance data

Statistical analyses were carried out with Statistical Package for the Social Sciences (SPSS), version 28 (for Windows; SPSS Inc., Chicago, IL, USA). Demographic and psychological data were analysed with analysis of variance and *post hoc t*-tests adjusted for multiple comparisons. Chi-squared and Fisher exact tests were used to analyse categorical demographic/psychological variables. Finally, ANCOVA was used to examine group differences in mean reaction time and response accuracy for each emotion, controlling for RSLE, age onset and duration of early-life stress. *Post hoc t-*tests were conducted pairwise between groups and Bonferroni correction was applied for multiple comparisons.

## Results

### Participant characteristics

All participants reported no current/past psychiatric disorders, and the information was corroborated with the DSM-5 Cross-Cutting and the KSADS-PL interviews. Two participants from the peer victimisation group and one participant from the control group were excluded because of MRI motion artefacts, leaving a final sample of 114 participants (39 childhood maltreatment, 37 peer victimisation and 38 controls).

Groups did not differ significantly in age, gender, IQ and socioeconomic status. As expected, the childhood maltreatment and peer victimisation groups scored significantly higher than controls on the BDI, BAI, NAPAS negative affect, RSLE, SHI and SDQ emotional and total difficulties scales (*P* < 0.01), but lower than controls on NAPAS positive affect scale (*P* < 0.001); nevertheless, their depression and anxiety scores were still within normative range below the cut-offs for moderate severity on the BDI and BAI, respectively. The childhood maltreatment and peer victimisation groups did not differ from each other, except on the SDQ peer problems, where the peer victimisation group had the highest score. The childhood maltreatment group had significantly lower age of onset and longer duration of early-life stress than the peer victimisation group (*P* < 0.001) (Table 1).

**Table 1:** Demographic characteristics of 39 youths exposed to childhood maltreatment, 37 youths exposed to peer victimisation and 38 controls

Characteristic	Childhood maltreatment group (*n* = 39)		Peer victimisation group (*n* = 37)		Control group (*n* = 38)		Analysis^d^^,^^e^		Group comparisons
	Mean	s.d.	Mean	s.d.	Mean	s.d.	*F*(2,111)	*P*-value	
Age (years)^b^	20.0	1.63	20.0	1.85	20.0	1.65	0.01	Not significant	−
IQ	104.6	9.92	102.8	7.86	102.5	7.24	0.68	Not significant	−
Socioeconomic status^c^	15.4	3.90	17.1	3.67	16.3	3.31	1.29	Not significant	−
Recent stressful life events scale	1.36	1.27	1.41	1.32	0.37	0.71	10.1	<0.001	Peer victimisation, childhood maltreatment > controls
Beck Depression Inventory	8.87	6.93	10.8	9.26	3.16	3.69	12.2	<0.001	Peer victimisation, childhood maltreatment > controls
Beck Anxiety Inventory	7.31	8.06	9.76	10.3	2.47	3.47	8.48	<0.001	Peer victimisation, childhood maltreatment > controls
Negative and Positive Affect Scale									
Negative affect	11.8	3.81	12.7	5.85	8.42	2.86	10.2	<0.001	Peer victimisation, childhood maltreatment > controls
Positive affect	17.8	4.68	17.7	4.49	23.1	3.13	21.1	<0.001	Peer victimisation, childhood maltreatment < controls
Strengths and Difficulties Questionnaire									
Emotional problems	3.82	2.11	4.19	2.56	2.34	1.67	7.89	0.001	Peer victimisation, childhood maltreatment > controls
Conduct problems	1.69	1.51	1.89	1.52	1.18	1.06	2.63	(0.07)	(Peer victimisation > controls)
Hyperactivity	4.13	2.52	3.84	2.51	2.76	2.21	3.39	0.04	Childhood maltreatment > controls
Peer problems	2.38	1.57	3.32	1.86	1.58	1.39	11.0	<0.001	Peer victimisation > childhood maltreatment, controls
Prosocial	7.33	2.22	7.70	2.00	8.53	1.47	3.87	0.02	Peer victimisation, childhood maltreatment < controls
Total difficulties score	12.0	5.06	13.2	6.27	7.87	4.40	10.7	<0.001	Peer victimisation, childhood maltreatment > controls
Childhood Trauma Questionnaire (CTQ)								CTQ severity classification	
Physical abuse	14.3	3.73	6.54	1.46	5.21	0.53	167.8	<0.001	Childhood maltreatment > peer victimisation, controls
Emotional abuse	16.9	3.89	8.00	2.17	5.82	1.09	187.3	<0.001	Childhood maltreatment > peer victimisation, controls
Physical neglect	9.54	2.83	6.38	2.03	5.68	1.12	36.2	<0.001	Childhood maltreatment > peer victimisation, controls
Emotional neglect	16.4	4.27	9.46	3.36	6.87	2.28	80.7	<0.001	Childhood maltreatment > peer victimisation, controls
Total score	57.2	10.2	30.4	6.66	23.6	3.61	223.5	<0.001	Childhood maltreatment > peer victimisation, controls
Revised Peer Experience Questionnaire									
Relational victimisation	1.33	1.45	8.66	2.67	0.53	0.83	218.8	<0.001	Peer victimisation > childhood maltreatment, controls
Overt victimisation	1.21	1.91	9.70	5.10	0.05	0.23	107.3	<0.001	Peer victimisation > childhood maltreatment, controls
Reputational victimisation	1.28	2.15	9.00	2.73	0.16	0.50	212.6	<0.001	Peer victimisation > childhood maltreatment, controls
European Cyberbullying Intervention Project Questionnaire	1.38	2.66	10.2	6.90	0.71	1.06	57.1	<0.001	Peer victimisation > childhood maltreatment, controls
							*F*(1,74)	*P-*value	
Age at onset of childhood maltreatment or peer victimisation (years)	6.36	2.64	10.6	2.41	−	−	53.2	<0.001	Childhood maltreatment < peer victimisation
Duration of childhood maltreatment or peer victimisation (years)	8.92	3.58	4.07	1.66	−	−	56.5	<0.001	Childhood maltreatment > peer victimisation
	*n*	%	*n*	%	*n*	%	*χ^2^*	*P-*value	Group comparisons
Gender^f^							0.06	Not significant	−
Male	16	41	15	41	16	42			
Female	23	59	22	59	22	58			
Ethnicity^f^:							7.27	Not significant	−
Chinese	33	85	30	81	36	94			
Malay	4	10	2	5	1	3			
Indian	2	5	5	14	1	3			
Self-Harm Inventory^a^^,^^f^	17	44	15	41	0	0	28.5	<0.001	Peer victimisation, childhood maltreatment > controls

a. Self-harm was determined by an answer of yes on at least one self-harm item on the Self-Harm Inventory.

b. The age range was 17–21 years.

c. The socioeconomic status total score ranges from 6 to 26, with higher values indicating higher status.

d. Tests adjusted for multiple comparisons.

e. The values in parentheses are marginally statistically significant.

f. The Fisher exact test was used.

### Task performance

There were no significant group differences on mean reaction times and performance accuracy for each emotion condition (Supplementary Table 1).

### Brain activation

#### Motion

Multivariate analyses of variance showed no significant group differences in maximum translation (Wilks’ Lambda *F*(6,226) = 0.73, *P* = 0.63) or maximum rotation (Wilks’ Lambda *F*(6,226) = 0.92, *P* = 0.48) parameters.

#### Group differences for emotion conditions

Within-group activations are shown in Supplementary Fig. 2. Groups differed significantly for the disgust–happy contrast only. The significant group effects were not driven by differences in happy processing, as there were no group differences for the happy contrast relative to fixation or implicit baseline.

For the ROI analysis, there were significant group differences in the right amygdala (*F*(2,108) = 3.48, *P* = 0.03) and marginal differences in the bilateral posterior insula (right: *F*(2,108) = 2.98, *P* = 0.05; left: (*F*(2,108) = 2.71, *P* = 0.07). Relative to controls, both childhood maltreatment and peer victimisation groups had significantly lower activation in the right amygdala (childhood maltreatment: *P* = 0.01; peer victimisation: *P* = 0.02) and bilateral posterior insula (childhood maltreatment: left: *P* = 0.02, right: *P* = 0.02; peer victimisation: left: *P* = 0.03, right: *P* = 0.02); the reduced left insular activation was furthermore related to increased self-harm within the childhood maltreatment group (*r*_pb_ = −0.23, *P* = 0.02; Supplementary Fig. 4(b)). There were no significant differences between the childhood maltreatment and peer victimisation groups.

At the whole-brain level, the childhood maltreatment group had significantly lower activation than controls in a cluster comprising predominantly bilateral hippocampus/hippocampal, thalamus, striatum, precuneus/posterior cingulate (PCC), inferior temporal, fusiform/lingual and cerebellar regions extending into the right amygdala, (posterior) insula and middle temporo-occipital regions (cluster 1), which was negatively associated with emotional problems within the control group, albeit at a marginal level (*r* = −0.29, *P* = 0.07; Supplementary Fig. 3); a cluster of left temporal and visual occipital regions (cluster 2); and a cluster of sensory processing regions, including the paracentral, pre-/postcentral and supplementary motor area (cluster 3), which was furthermore associated with increased self-harm in maltreated youths (*r*_pb_ = −0.33, *P* = 0.04; Supplementary Fig. 4(a)).

**Fig. 1:**
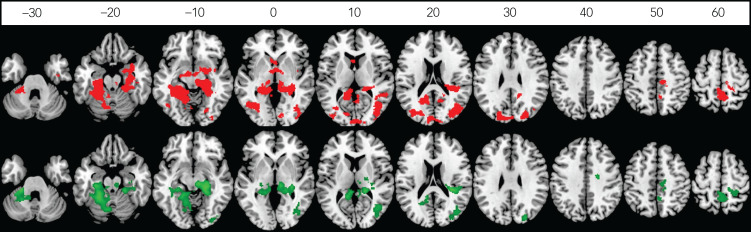
Between-group differences in brain activation of disgust versus happy contrast in childhood maltreatment compared with controls (red), and in peer victimisation compared with controls (green). Axial sections showing decreased activation of disgust versus happy contrast in 39 maltreated young people compared with 38 controls (red) and in 37 bullied young people compared with 38 controls (green) (*P* < 0.05 family-wise error rate corrected at the cluster level). Axial slices are marked with the *z*-coordinate as distance in millimetres from the anterior–posterior commissure. The right side of the image corresponds to the right side of the brain.

The peer victimisation group also exhibited reduced activation in these three clusters relative to controls in the whole-brain analysis. Furthermore, when BOLD responses of the above clusters were extracted for planned comparison with the peer victimisation group by using ANCOVA controlling for RSLE, age onset and duration of early-life stress, the peer victimisation group also exhibited reduced activation in these clusters relative to controls only (Table 2, Fig. 1).

**Table 2:** Group differences in brain activation

Brain regions	Cluster level			
	Peak MNI coordinates (*x, y, z*)	Number of voxels	*P-*value^a^	Group comparisons^b^
Bilateral fusiform, parahippocampal, hippocampus, inferior temporal gyri, thalamus, striatum, lingual, precuneus, cuneus, posterior cingulate cortices, cerebellum lobule IV-V and vermis extending to right amygdala, (posterior) insula, middle and superior temporal, inferior, middle and superior occipital and supramarginal gyri	−24, −40, −16	8992	<0.001	Childhood maltreatment, peer victimisation < controls
Left inferior and middle temporal gyri, and inferior, middle, superior occipital gyri extending to bilateral fusiform, lingual cuneus and calcarine	−32, −68, 4	1802	<0.001	Childhood maltreatment, peer victimisation < controls
Bilateral paracentral lobules and precuneus extending to right supplementary motor area, postcentral and precentral gyri	−2, −38, 62	1029	0.014	Childhood maltreatment, peer victimisation < controls

MNI, Montreal Neurological Institute.

a. Family-wise error rate-corrected *P*-values.

b. Group differences in brain activation were conducted with number of recent stressful life events, age onset and duration of early-life stress exposure as covariates.

## Discussion

To our knowledge, this is the first fMRI study on emotional processing in childhood maltreatment and peer victimisation that has used a sizable community youth sample free from psychopathology, medications and drug misuse, and controlled for the number of recent stressors and the timing and duration of early-life stress. This is essential to elucidate the effects of early-life stress from the confounding effects associated with current stressors, psychiatric comorbidities, medications and drug use.^7^

There were no significant group differences in task performance, and groups differed in brain activation during disgust processing only. For the ROI analysis, both childhood maltreatment and peer victimisation groups had lower activation in the right amygdala and bilateral posterior insula than controls, with reduced left insular activation furthermore being related to increased self-harm in maltreated individuals. Compared with controls, at the whole-brain level, both childhood maltreatment and peer victimisation groups had lower activation in a cluster of predominantly bilateral limbic-thalamic-striatal, precuneus/PCC and fusiform/lingual regions, which was negatively associated with emotional problems in controls, as well as a cluster of somatosensory regions that was associated with increased self-harm in maltreated individuals. The reduced activation of key emotion and sensory processing regions during disgust processing may possibly serve as an avoidance coping mechanism to protect youths who experience early-life stress from distressing emotional responses by blocking the processing of aversive disgust facial expressions, thereby enabling them to achieve equivalent performance with their counterparts who do not experience early-life stress. However, this may increase their vulnerability for affective psychopathology later in life.

Most earlier studies have focused primarily on negative emotions of fear and anger, whereas disgust has been the most understudied of all emotions.^37^ Nonetheless, there is a growing recognition of the role of disgust as a central emotion in trauma-related disorders, and that it can lead to self-disgust.^38^ Disgust is a basic emotion and represents an evolutionary adaptive defensive-avoidance response.^37^ Disgust facial expressions signal interpersonal rejection and have been linked to feelings of self-disgust and debasement,^39^ which is a potential mechanism underlying the association between childhood maltreatment and self-harm/suicide risk.^20^

Recent event-related potential findings reported that childhood maltreatment is associated with an automatic early vigilance and a subsequent attentional avoidance of disgust faces in healthy young adults, where attentional avoidance may be a coping strategy adopted by the maltreated individuals to downregulate their experience of disgust and avoid conflicts.^40^ Therefore, childhood interpersonal trauma such as childhood maltreatment and peer victimisation, which often involve repeated exposure to facial disgust in others signalling rejection, may affect the victim's processing of disgust and further heighten their vulnerability to affective psychopathology, possibly via feelings of (self-) disgust.

Besides its critical role in interoceptive processes, the insula is also relevant in the processing of negative emotions including disgust and fear,^41^ which are frequently experienced in childhood interpersonal trauma. Although the anterior part is usually implicated, mounting research also suggests the involvement of the posterior insula in aversive emotion and sensory processing.^42^ The posterior insula, which has strong projections to the amygdala and is a major cortical convergence site that integrates signals from within and outside the body, has also been implicated as a neuroanatomical hub underlying various psychiatric conditions.^43^

Aberrant amygdala and insula activations have been found to mediate atypical emotion processing of fear and anger in childhood maltreatment^3,10^ and peer victimisation.^17^ Recent large-scale transdiagnostic studies also implicated the role of insula in childhood emotional trauma.^44^ In addition, a recent meta-analysis found that youths with self-injurious behaviours exhibited insula functional alterations, which may underlie their core symptoms of altered emotion processing and regulation.^45^ Thus, the current findings of atypical limbic activation during disgust processing extends earlier findings of altered threat processing in childhood trauma, and underscores the importance of examining disgust processing in early-life interpersonal stress and self-harm. Furthermore, given that individuals with a history of self-injurious behaviours and suicide have higher pain tolerance than controls,^46^ the reduced activation of the limbic and somatosensory regions, which are also part of the pain modulatory brain networks,^47^ may potentially modulate pain perceptions and increase self-harm/suicide risk by lowering pain sensitivity in maltreated youths.

Converging evidence underscores the link between childhood trauma and atypical development of the visual sensory systems that convey adverse experiences. For instance, morphometric studies reported reduced lingual GMV^6^ and reduced fractional anisotropy of the inferior longitudinal fasciculus and inferior frontal-occipital fasciculus in childhood maltreatment, where the reduced fractional anisotropy values were associated with greater severity and longer duration of abuse.^48^ A recent meta-analysis further suggests that disruption in these visual-emotional processing tracts may underpin the emotional problems commonly observed in childhood trauma.^8^ Reduced somatosensory GMV in childhood maltreatment has also been reported in a meta-analysis of structural MRI studies of childhood maltreatment^7^ and in recent large-scale transdiagnostic studies.^49^ Thus, the current findings of reduced activation of the visual and somatosensory regions in youths who experience early-life stress, together with structural abnormalities in these regions reported in earlier studies, suggest that the visual sensory systems may be altered by early-life stress exposure.

The PCC and precuneus form part of the default mode network and are involved in self-referential processing and mental representation, with increased activations being associated with distraction from task performance.^50^ It has been purported that the PCC mediates experiences of identifying with attributes of ourselves.^51^ Hence, the observed reduced activation of the PCC/precuneus by our early-life stress groups may suggest an attempt to inhibit self-related thinking of highly relevant emotions that are potentially associated with oneself, such as disgust, to stay focused on the current task. PCC/precuneus are also key areas of visuospatial attention^52^ and a reduced activation could be attributable to reduced attention or suppression of attention to negative emotional stimuli. In line with this, a recent study found that chronically bullied youths exhibited lower precuneus activation than controls in response to viewing cyberbullying stimuli, thereby signalling habituation to the aversive stimuli.^53^ Thus, the reduced precuneus activation observed in our early-life stress groups may also reflect their habituation to facial disgust.

Both childhood maltreatment and peer victimisation groups achieved comparable task performance as controls, but with a shared under-recruitment of task-relevant brain regions during disgust processing; hence, differences in the activated regions cannot be attributed to performance effects. Moreover, we note that our childhood maltreatment and peer victimisation youths represent high-functioning community youths with comparable IQ and socioeconomic status to the controls. Thus, the atypical brain activation patterns and lack of performance deficits in childhood maltreatment and peer victimisation groups may suggest that the two early-life stress groups employed an adaptive or compensatory neural strategy to achieve comparable task performance.

The shared atypical activation patterns observed in both early-life stress groups, who also had comparable psychological and socioeconomic status scores, underscore the detrimental effects of peer victimisation and suggest that negative peer relationships may be as harmful as abusive caregiving. More research attention on the neural correlates of peer victimisation is warranted. Furthermore, it is intriguing to note that reduced activation of key emotion and sensory processing regions was associated with increased self-harm in maltreated individuals only. It has been postulated that feelings of self-disgust and debasement, which have been linked to disgust facial expressions,^39^ may be a potential mechanism underlying increased self-harm/suicide risk in childhood maltreatment.^20^ Although we did not directly test for self-disgust and debasement in the current study, a similar mechanism might be at work. Given that harsh parenting exhibited incremental importance over and above peer victimisation on youths’ self-cognition,^22^ we propose that, for our childhood maltreatment group, growing up with constant exposure to disgust expressions from caregivers might have detrimentally affected their internal self-schemas, leading to feelings of worthlessness and perceived burdensome, and thereby increasing their vulnerability for self-harm/suicide. Indeed, research has shown that children internalise expectations from parents in their construction of self-concept, and chronic negative parental feedbacks and rejection are linked to internalising symptoms.^54^ Thus, we postulate that prolonged exposure to disgust faces, which essentially signal rejection, especially from parents from whom a child typically derives a sense of self-worth during early critical developmental stages, may result in the eventual internalisation of a repulsive self-concept. An inward disgust response to one's self-identity may thus potentiate feelings of perceived burdensome and thwarted belongingness, which further heightens self-harm/suicide risk.

Finally, we did not observe atypical activation during anger and fear possessing, possibly because of our more resilient community-based sample compared with earlier studies. The maltreated and bullied youths in our sample are healthy and high-functioning individuals with similar IQ and socioeconomic status as controls, and have depressive and anxiety scores within normative ranges, albeit higher than controls. Therefore, instead of viewing adverse early experiences as factors leading to general developmental deficits *per se*, the current findings of reduced recruitment of key emotion and sensory processing regions may reflect the brain's attempt to cope by blocking the processing of aversive stimuli and functionally adapt to growing up in hostile environments in ways that promote survival. Nonetheless, we appreciate that prolonged neural overmodulation during disgust processing, despite meeting the immediate contextual demands, may have negative long-term consequences such as hindering the development of appropriate emotion regulation skills, potentially paving the way to affective disorders and self-injurious behaviours.

This study is not without its limitations. First, it is cross-sectional and the findings are still correlational. Second, the use of retrospective self-report data may be subject to recall biases. Third, the results may not be generalisable to the childhood sexual abuse population. Nevertheless, strengths of this study are that all participants were free from psychopathology, medications and drug misuse; their current stressors were assessed and controlled for; the early adverse experiences were carefully substantiated by semi-structured interviews and we used dynamic stimuli that are more realistic representation of real-life emotional expressions. The slight discrepancy in the ROI and whole-brain findings could be because the ROIs were examined without correction for multiple comparisons, whereas the whole-brain analysis was performed using rigid statistics (family-wise error rate correction). Nonetheless, our whole-brain results reinforced the ROI findings of atypical limbic activation in our early-life stress groups, particularly in the right amygdala and posterior insula. Finally, although the generalisability of the results may be restricted to the ‘more resilient’ portion of community youths without any psychiatric disorders, the current findings underscore that individuals exposed to early-life stress do show neural alterations compared with their unexposed counterparts, even in the absence of reported psychopathology.

## Supporting information

Lim et al. supplementary material Lim et al. supplementary material

## Data Availability

The data that support the findings of this study are available from the corresponding author, L.L., upon reasonable request.
