# Supplementary material for: Common neural correlates of disgust processing in childhood maltreatment and peer victimisation
Source: BJPsych Open. 2024 Oct 28;10(6):e185. doi: 10.1192/bjo.2024.767 (PMC11698183; doi:10.1192/bjo.2024.767)
Supplement: Lim et al. supplementary material [file S2056472424007671sup001.docx]

**SUPPLEMENTARY INFORMATION**

***Incident Findings***

The study was conducted solely for research and not for medical diagnostic purposes, though all MRI scans were reviewed by a neuro-radiologist for inconclusive signs or indications of possible medical conditions that may require further investigations. During informed consent taking, participants and/or their legal caregivers were given the option to be re-identified and notified in the case of incidental findings and their decisions would be respected. If they agreed, the participants and/or their legal caregivers would be notified in the event of an important incidental finding. In exceptional situations such as the discovery of a life-threatening incidental finding, legal caregivers of participants would be contacted to re-confirm their decision whether to learn more about the incidental finding. Participants and/or their caregivers were advised not to look to the study for information on the state of the participant’s health and for medical care and management, and were asked to consult their doctor for further investigation. There were no cases of incidental findings in this study.

***fMRI Data Acquisition and Analysis***

MRI data was acquired on a 3T Siemens MAGNETOM Prisma (Siemens Healthcare GmbH, Germany) scanner at NTU with a 64-channel head coil. In each of 50 non-contiguous planes parallel to the anterior-posterior commissure, 376 interleaved T2*-weighted MR images depicting Blood Oxygen Level Dependent (BOLD) contrast covering the whole brain were acquired with the EPI sequence (TR=1500ms, TE=30ms, flip angle 40°, isotropic voxel size 2.5mm^3^ and FOV 240mm). The first 10s of dummy scans were always discarded to minimise noise from scanner magnetisation instability. The MPRAGE sequence (TR=2200ms, TE=2.28ms, T1=994ms, 208 slices, isotropic voxel size 1mm^3^, flip angle 8° and FOV 260mm) was used to acquire the T1-weighted structural images.

Data were analysed using SPM12 (www.fil.ion.ucl.ac.uk/spm). Images were motion corrected with all images being realigned to the first scan in the time-series and then the mean image. After realignment, images were co-registered to the high-resolution EPI. All scans were normalised to standard space, using the EPI template, with the parameters derived from the high-resolution EPI and applied to the functional time series. Data were spatially smoothed using a kernel of 8mm full-width half-maximum (FWHM).

At the first level, a general linear model (GLM) approach was followed to allow for contrasts of each negative emotion condition against happy. Regressors were convolved with the canonical hemodynamic response function to model the blood oxygen level dependent (BOLD) signal. Regressors for events of interest were disgust, happy, fear, anger, neutral and fixation. First- and second-order movement parameters were also included in the model as regressors for events of no interest. A high-pass filter (128s) was applied to the data and first-order temporal autocorrelation was modelled. Weighted contrasts were used to test the effect of changes on BOLD signal during each negative emotion contrasted with happy for each individual. Given that individuals with histories of early trauma tend to perceive neutral faces as negative (Hart *et al.,* 2018), all negative emotions were contrasted with happy rather than neutral. Contrasting negative emotions with happy is also a common practice in fMRI studies of emotion processing (Hart *et al.,* 2018; McClure *et al*., 2007; Whalen *et al*., 2008; Klumpp *et al*., 2012). Furthermore, the neutral condition did not contain the same amount of facial movement as the emotion conditions and hence the happy condition is a better matched contrast as it controlled for motion perception.

**Table S1** Task performance of 39 young people exposed to childhood maltreatment, 37 young people exposed to peer victimisation and 38 controls

|  |  | **Childhood Maltreatment group**  **(N=39)** | | **Peer Victimisation group**  **(N= 37)** | | **Control group**  **(N=38)** | |  | **Analysis^a,b^** | | |
| --- | --- | --- | --- | --- | --- | --- | --- | --- | --- | --- | --- |
| **Emotion** | **Performance Measure** | **Mean** | **SD** | **Mean** | **SD** | **Mean** | **SD** |  | **F(2, 108)** | ***p* (corr.)** | **Between Groups** |
| Disgust | Mean reaction time (ms) | 774 | 122 | 765 | 168 | 785 | 112 |  | 0.49 | ns | - |
|  | Response accuracy (%) | 97.5 | 4.03 | 95.7 | 9.23 | 98.7 | 2.39 |  | 0.90 | ns | - |
| Happy | Mean reaction time (ms) | 681 | 96 | 699 | 113 | 699 | 93 |  | 0.33 | ns | - |
|  | Response accuracy (%) | 98.2 | 3.73 | 98.5 | 2.56 | 98.8 | 2.11 |  | 0.37 | ns | - |
| Fear | Mean reaction time (ms) | 756 | 147 | 768 | 172 | 781 | 134 |  | 0.32 | ns | - |
|  | Response accuracy (%) | 92.1 | 10.5 | 91.6 | 9.28 | 95.1 | 10.1 |  | 0.37 | ns | - |
| Anger | Mean reaction time (ms) | 777 | 170 | 792 | 185 | 780 | 138 |  | 0.48 | ns | - |
|  | Response accuracy (%) | 86.8 | 16.5 | 85.0 | 15.6 | 93.9 | 8.80 |  | 0.68 | ns | - |
| Neutral | Mean reaction time (ms) | 750 | 134 | 719 | 128 | 734 | 120 |  | 0.22 | ns | - |
|  | Response accuracy (%) | 86.9 | 13.9 | 90.9 | 6.60 | 92.3 | 7.40 |  | 1.30 | ns | - |

^a^ Adjusted for multiple comparisons.

^b^ Group differences in task performance were conducted with number of recent stressful life events (RSLE), age onset and duration of early-life stress exposure as covariates.

**Fig. S1** Image of amygdala, anterior and posterior insula masks


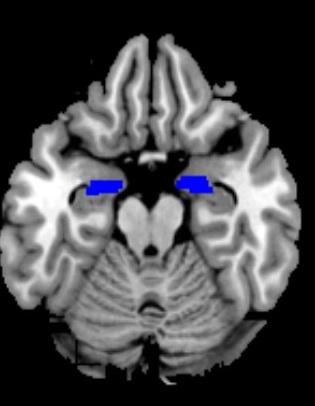

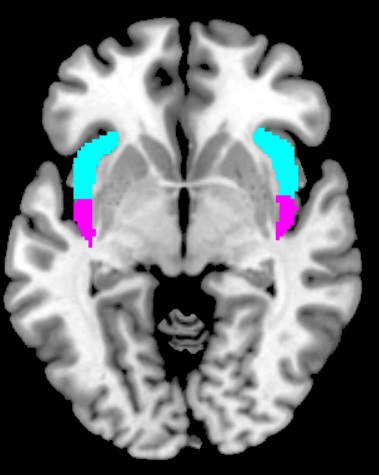


L

Anterior Insula

Posterior Insula

Amygdala

z=-20

z=-5

**Fig. S2** Within-group brain activation

***Disgust***

**CM:**

**PV:**

**C:**

**-30 -20 -10 0 10 20 30 40 50 60**

***
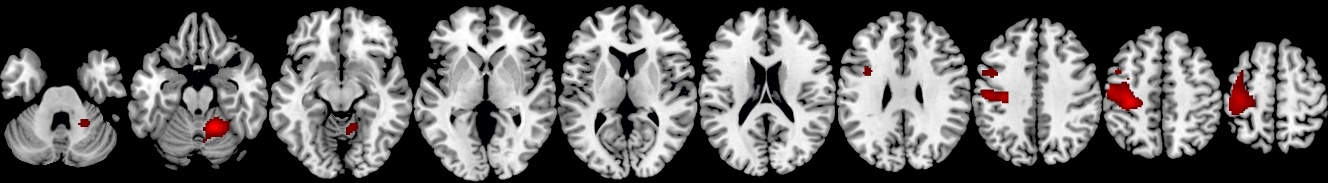
***

**
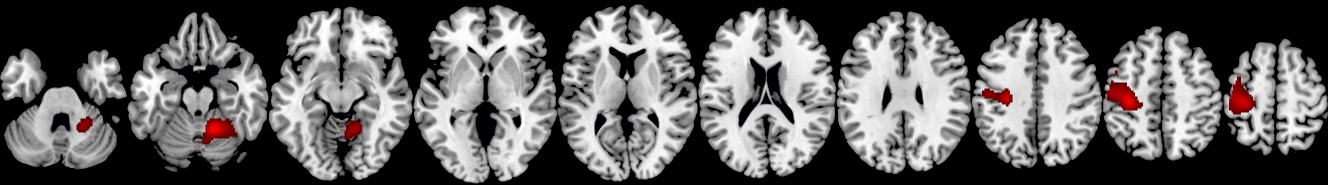
**

**
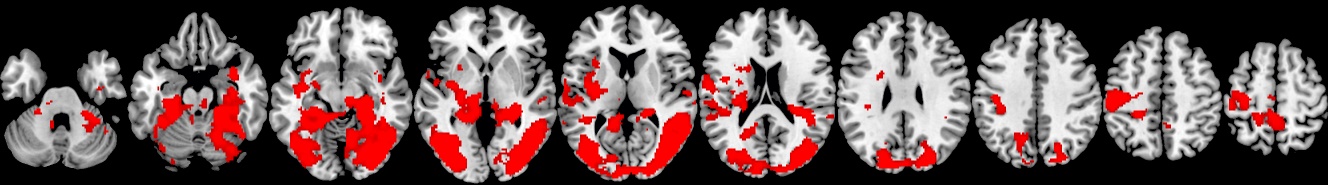
**

***Neutral***

**CM:**

**PV:**

**C:**

**-30 -20 -10 0 10 20 30 40 50 60**

***
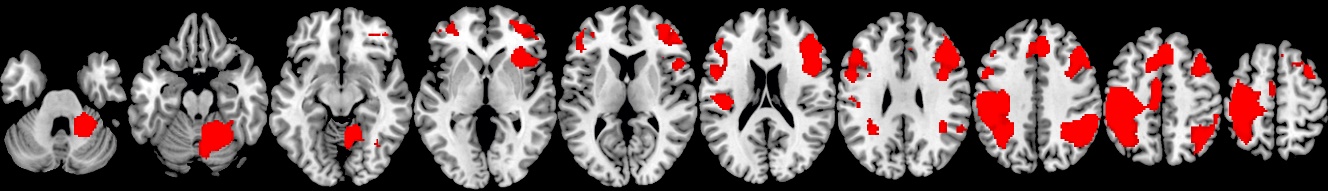
***

***
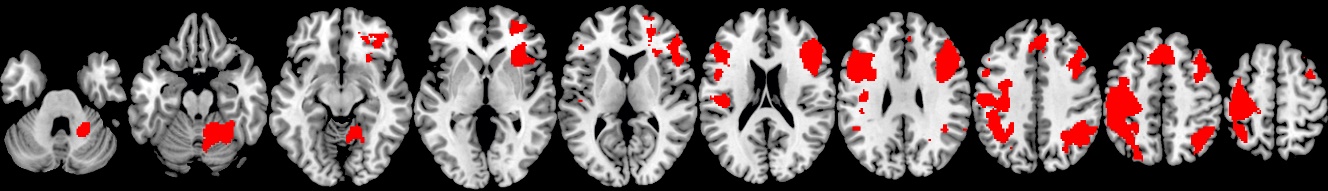
***

None

***Fear***

**CM:**

**PV:**

**C:**

**-30 -20 -10 0 10 20 30 40 50 60**

**
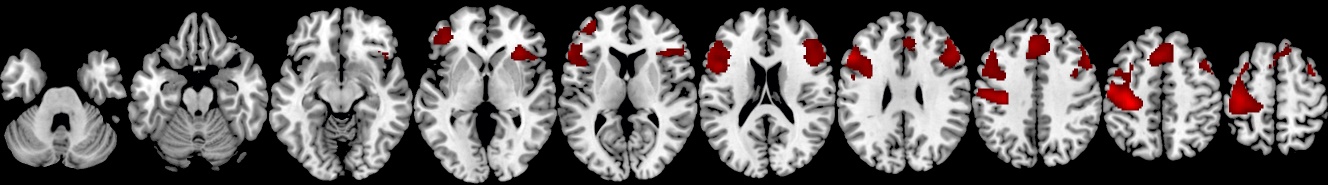
**

**
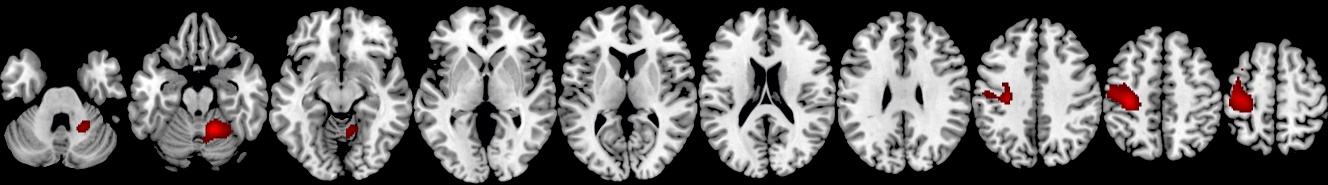
**

None

***Anger***

**CM:**

**PV:**

**C:**

**-30 -20 -10 0 10 20 30 40 50 60**

**
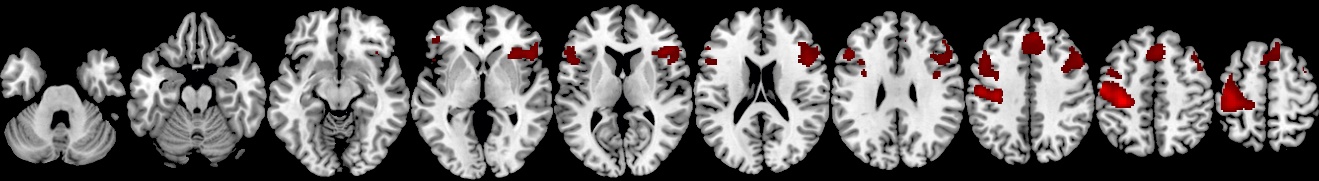
**


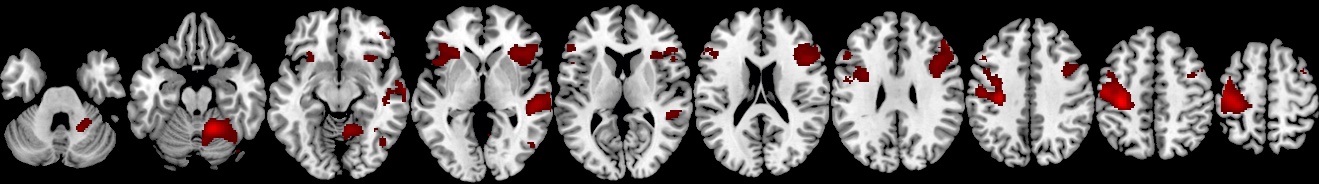


None

Axial sections of brain activation of each emotion versus happy contrasts for 1) childhood maltreatment (CM) group, 2) peer victimisation (PV) group and 3) control (C) group, *p*<0.05, FWE-corrected at cluster level. Axial slices are marked with the z coordinate as distance in millimetres from the anterior–posterior commissure. The right side of the image corresponds to the right side of the brain.

**Fig. S3** Scatterplot of negative correlation between mean beta values of cluster 1 and SDQ emotional problems within the control group

**Fig. S4** Boxplots of mean beta values of **(a)** cluster 3 and **(b)** left posterior insula for self-harm within the childhood maltreatment group

**(a) (b)**


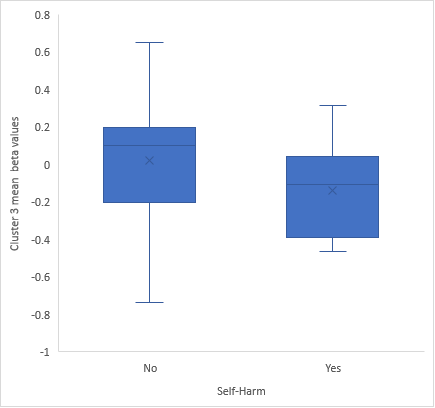

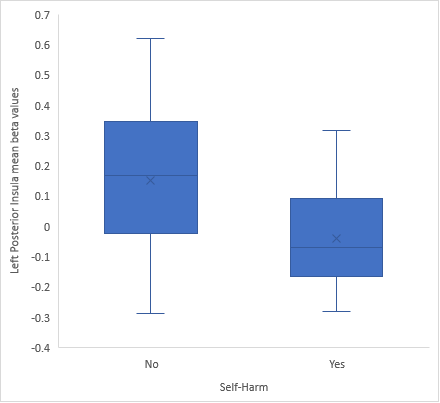


**References**

Hart H, Lim L, Mehta M, Simmons A, Mirza K, Rubia K (2018). Altered fear processing in adolescents with a history of severe childhood maltreatment: An fMRI study. *Psychological Medicine* 48, 1092-1101.

Klumpp H, Angstadt M, Phan, KL (2012). Insula reactivity and connectivity to anterior cingulate cortex when processing threat in generalised social anxiety disorder. *Biological Psychology* 89, 273-276.

McClure EB, Adler A, Monk CS, Cameron J, Smith, S, Nelson EE, Leibenluft E, Ernst M, Pine DS (2007). fMRI predictors of treatment outcome in pediatric anxiety disorders. *Psychopharmacology* 191, 97-105.

Whalen PJ, Johnstone T, Somerville LH, Nitschke JB, Polis S, Alexander AL, Davidson RJ, Kalin, NH (2008). A functional magnetic resonance imaging predictor of treatment response to venlafaxine in generalised anxiety disorder. *Biological Psychiatry* 63, 858-863.
